# Disproportionate cardiac hypertrophy during early postnatal development in infants born preterm

**DOI:** 10.1038/pr.2017.96

**Published:** 2017-05-24

**Authors:** Christina Y L Aye, Adam J Lewandowski, Pablo Lamata, Ross Upton, Esther Davis, Eric O Ohuma, Yvonne Kenworthy, Henry Boardman, Samuel Wopperer, Alice Packham, Satish Adwani, Kenny McCormick, Aris T Papageorghiou, Paul Leeson

**Affiliations:** 1 Oxford Cardiovascular Clinical Research Facility, Division of Cardiovascular Medicine, University of Oxford, Oxfordshire, UK; 2 Department of Biomedical Engineering, King’s College London, London, UK; 3 Centre for Statistics in Medicine, University of Oxford, Oxford, Oxfordshire, UK; 4 Department of Paediatrics and Neonatology, John Radcliffe Hospital, Oxford, Oxfordshire, UK; 5 Nuffield Department of Obstetrics & Gynaecology, University of Oxford, Oxford, Oxfordshire, UK

## Abstract

**Background:**

Adults born very preterm have increased cardiac mass and reduced function. We investigated whether a hypertrophic phenomenon occurs in later preterm infants and when this occurs during early development.

**Methods:**

Cardiac ultrasound was performed on 392 infants (33% preterm at mean gestation 34±2 weeks). Scans were performed during fetal development in 137, at birth and 3 months of postnatal age in 200, and during both fetal and postnatal development in 55. Cardiac morphology and function was quantified and computational models created to identify geometric changes.

**Results:**

At birth, preterm offspring had reduced cardiac mass and volume relative to body size with a more globular heart. By 3 months, ventricular shape had normalized but both left and right ventricular mass relative to body size were significantly higher than expected for postmenstrual age (left 57.8±41.9 vs. 27.3±29.4%, *P*<0.001; right 39.3±38.1 vs. 16.6±40.8, *P*=0.002). Greater changes were associated with lower gestational age at birth (left *P*<0.001; right *P*=0.001).

**Conclusion:**

Preterm offspring, including those born in late gestation, have a disproportionate increase in ventricular mass from birth up to 3 months of postnatal age. These differences were not present before birth. Early postnatal development may provide a window for interventions relevant to long-term cardiovascular health.

Worldwide, ~1 in 10 infants are born preterm, and owing to improved maternal–fetal and neonatal intensive care the majority now survive to adulthood (1). However, this survival may be at the expense of long-term cardiovascular sequelae. Birth triggers a switch in cardiomyocytes from a fetal hyperplastic to hypertrophic growth pattern (2, 3). In experimental preterm models, exposure of immature cardiomyocytes to the high-resistance, relatively hyperoxic, postnatal, arterial circulation results in their significant hypertrophy (2, 3). This may also occur in humans, as adults born preterm have a unique cardiac shape, as well as significantly increased cardiac mass (4). Furthermore, the severity of the changes in adulthood are proportional to the degree of prematurity (4, 5), and can be modified by exposures during this early developmental period (6, 7).

To test the hypothesis that there is evidence of postnatal changes to cardiac performance in response to the *ex utero* environment in the preterm infant, we performed serial echocardiograms at birth and 3 months of age to assess differences in maturational patterns of cardiac structure and function between preterm and term infants. We then compared the changes in cardiac mass, volumes, and shape with those expected during term pregnancies from early gestation, taking account of variation in body growth and other perinatal factors.

## Methods

### Study Overview

Between 2011 and 2015, mothers being cared for by the Oxford University Hospitals NHS Foundation Trust were identified by their clinical care team and invited to take part in one or more of a portfolio of studies coordinated by the Oxford Cardiovascular Clinical Research Facility. These studies were designed to investigate the impact of pregnancy complications on cardiovascular development during gestation and after birth.

### Neonatal Cohort Selection and Imaging Assessments

**Figure 1:**
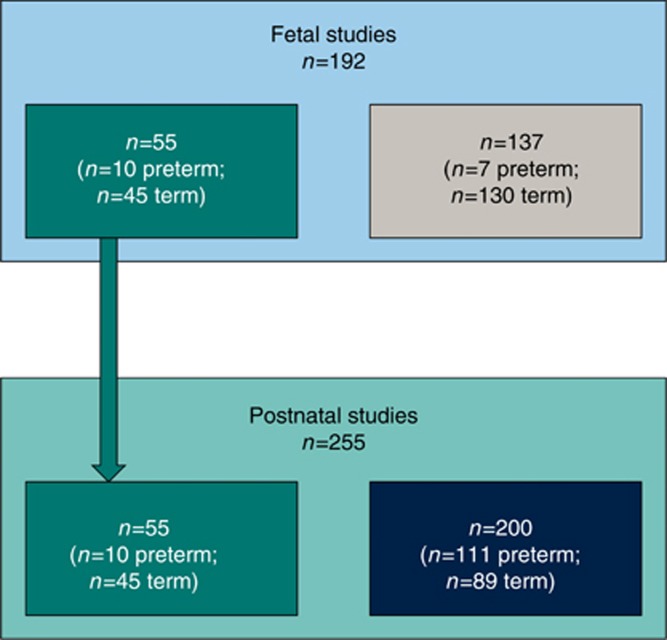
Flow diagram of study recruitment. A total of 392 individuals took part in the study for echocardiography imaging. One hundred and ninety two individuals were recruited during gestation (fetal studies) to undergo echocardiography scans, with *n*=55 (*n*=10 preterm; *n*=45 term, shown in teal) going on to take part in the postnatal studies. An additional *n*=200 individuals were recruited at birth (shown in dark blue) to take part in birth and 3-month postnatal age echocardiography scans (total *n*=255 postnatal studies, *n*=121 preterm; *n*=134 term).

A stratified recruitment approach to ensure balanced representation of preterm and term birth, as well as hypertensive and normotensive pregnancies, was used through the EPOCH (Effect of Preterm birth and pregnancy Hypertension on Offspring Cardiovascular Health) study (South Central Berkshire Research Ethics Committee ref. 11/SC/0006, UKCRN/clinical trials ref. NCT01888770) (8). On designated days of the week, consecutive women who had delivered preterm and/or who had experienced a hypertensive pregnancy were approached to take part in the study. A subgroup of women who had experienced a term, normotensive pregnancy delivering on the same day were also approached to serve as a control group. In total, 255 infants were recruited to the neonatal cohort and underwent echocardiography soon after birth and at 3 months of age (Figure 1). The phenotypic switch in cardiomyocytes is thought to take place in the first two weeks of postnatal development (9). We therefore chose 3 months of age as the timing for our follow-up assessment, as at this point even the most preterm in our cohort would be a few weeks post term-equivalent age. The majority of preterm infants studied were born moderate to late preterm, between 32 and 36 weeks (102/121=84%).

### Fetal Cohort Selection and Imaging Assessments

The final 55 mothers from our neonatal cohort were recruited antenatally and fetal echocardiography performed starting at 15 weeks of gestation and at multiple time points until delivery (Figure 1). Fetal echocardiography was also performed in a cohort of 137 uncomplicated pregnancies within the INTERBIO-21st study (South Central—Oxford C Research Ethics Committee ref. 08/H0606/139). This was a longitudinal study in which women were attended every 4 weeks during pregnancy for an obstetric ultrasound scan. On designated days, all mothers attending an assessment had fetal echocardiography performed as part of their scan. Therefore, some fetuses underwent multiple echocardiograms, whereas others only had measurements at a single time point. Of the *n*=192 fetuses scanned, 110 fetuses were scanned once; 43 were scanned twice; 21 were scanned three times; 12 were scanned four times; and six were scanned five times.

All mothers gave written informed consent and assent for involvement of their children, including permission to access maternal and offspring clinical records. Singletons and multiples were included. Mothers below the age of 16 years were excluded from the study as were those with chronic cardiovascular conditions prenatally, including hypertension, although those who suffered from hypertensive disorders of pregnancy were included. Infants were excluded if they had evidence of any severe malformations, congenital cardiovascular disease, chromosomal abnormalities, or genetic disorders, but those with persistent features of a fetal circulation at birth, i.e., patent ductus arteriosus (PDA) and/or atrial septal defect (ASD), were included.

### Anthropometric and Blood Pressure Measurements

Gestational age at the time of measurements was calculated relative to gestational age defined at first trimester ultrasound. At both the birth and 3-month assessments, weight was measured using digital scales (Charder Model MS4200) to the nearest 0.01 kg with the infant fully naked. During gestation, head circumference was taken from a cross-sectional view of the fetal head at the level of the thalami as close as possible to the horizontal as previously described (10) and the average of three measurements used. Postnatally, head circumference was measured with a tape measure to the nearest millimeter. *Z*-scores for weight were calculated using the international standard size at birth reference charts from the INTERGROWTH-21st Project (11, 12) using their online application (https://intergrowth21.tghn.org/global-perinatal-package/intergrowth-21st-comparisonapplication/).

Data collection methods and clinical characterization have previously been reported (8) and are included in the Supplementary Methods online.

### Echocardiography

Fetuses were scanned on a Philips HD9 with a C6-3 curved-array transducer. At birth, a Phillips CX50 was used with a 3-month follow-up on a Philips iE33 with an S12-4 transducer. At each time point, a 2D transthoracic echocardiography protocol that included acquisition of a four-chamber view optimized for the LV was performed according to standard guidelines (13). Postnatal measures were performed in a temperature-controlled room, with the infant at rest in a semi-recumbent position at 45 degrees, either in their mother’s arms or in a crib. Frame rates were maximized and multiple images were acquired for off-line selection of high-quality loops. Standard methods for quantification of volume and mass of the left ventricle were performed (14) based on linear measures of wall thickness using standard approaches (15). In addition, we used TomTec Image Arena 4.6 to create automated estimates of LV mass based on endocardial and epicardial borders defined in multiple four-chamber cine loops. An adaptation of the method was applied to the RV, as previously reported (16). Body size correction for measures during gestation and in analyses from gestation through postnatal development was based on adjustment for head circumference, as this provided a directly quantified indicator of growth, known to be accurate in the antenatal and postnatal period (17). In addition, for neonatal measures, ventricular mass and volumes were adjusted for body size based on estimated body surface area, using the Boyd formula (18), and these values are reported as mass or volume index. Morphology of the LV in the four-chamber view was analyzed by construction of a computational statistical shape model, through adaptation of a technical approach previously described for magnetic resonance images (19, 20). Detailed methods and our laboratory inter- and intra-observer variability for measures are described in the Supplementary Methods.

Investigators performing the assessments were not blinded to participant group, but those involved in image analysis were.

### Statistical Analysis

Statistical analysis was performed using SPSS Version 20 (IBM, Armonk, New York, NY) and GraphPad Prism Version 6.0 (La Jolla, CA). In addition, STATA, Version 11.2, software (StataCorp LP, College Station, TX) was used to analyze developmental trajectories and cardiac shape analysis was performed with Matlab (Mathworks, Natick, MA). Values are presented as mean±SD unless stated otherwise.

To compare mean differences in cardiac size postnatally, Student’s *t*-test and Mann–Whitney *U* test were used as appropriate. Tests of associations were performed using the χ^2^ test. Linear regression models were performed with a forced entry method. Pearson correlations (*r*) were used for bivariate associations, and unstandardized regression coefficients (*B*) were used for bivariate and multivariate regression models. Variables were selected from bivariate regression models to be included in multivariate regression models. The sample size *n*=134 for term offspring and *n*=121 preterm offspring provided us with 80% power at a significance level of *α*=0.05 to detect a difference of at least 0.38 SDs between groups.

To further explore the timing of mass and volume changes in preterm infants, we studied trajectories of cardiac development using the combined data set of echocardiographic measures of LV and RV mass at different time points during gestation and postnatal development. As volumes pre- and postnatally would not be comparable because of circulatory changes at birth, we limited combined gestation and postnatal analyses to mass. Smoothed centiles of right and left ventricular mass, left and right EDV, and ratio of mass as a function of HC according to gestational age were constructed using fractional polynomials. To account for body size differences between preterm and term infants at different time points, we adjusted the left and right ventricular mass for head circumference (i.e., left ventricular mass/HC) on the basis that this measure would be the most consistently and accurately quantifiable body measure throughout gestation and postnatal development rather than weight or body surface area. This is because weight cannot be directly or accurately measured in gestation and therefore pre- and postnatal values cannot be directly compared. In addition, equations for body surface area have not been validated for fetuses. Where appropriate, we applied a multilevel, linear regression analysis to account for repeated measures (21), but there were no significant differences when compared with analyses that did not account for the hierarchy of the data. LV and RV mass, volume, and ratio of mass as a function of HC exhibited a non-normal distribution; therefore, the data were log-transformed (natural log) to stabilize variance and transform the data to normality. Goodness-of-fit assessment incorporated a visual inspection of the quantile–quantile (q–q) plot of the residuals, and a plot of fitted *z*-scores across gestational ages.

Analysis of shape variation between preterm and term infants was performed by a non-paired *t-*test. Shape differences were first tested in each Principal Component Analysis mode under three conditions: (1) at birth, (2) at follow-up, or (3) by their growth. Then, the Principal Component Analysis modes, which accounted for the most variation within the experimental sample, were combined through a LDA in an attempt to identify the morphological signature of a premature birth. Cross-validation (leave-1 out) was used to test the generality of differences found in the LDA, and to select the optimal set of Principal Component Analysis modes to be combined to differentiate groups.

*P*-values<0.05 were considered statistically significant.

## Results

### Cohort Characteristics

Maternal and offspring demographic and anthropometric characteristics in the preterm and term groups are presented in Table 1. Characteristics between groups and available data sets were similar and are described in full in Supplementary Table S1.

### Postnatal Increase in Ventricular Mass in Preterm-Born Infants

**Figure 2:**
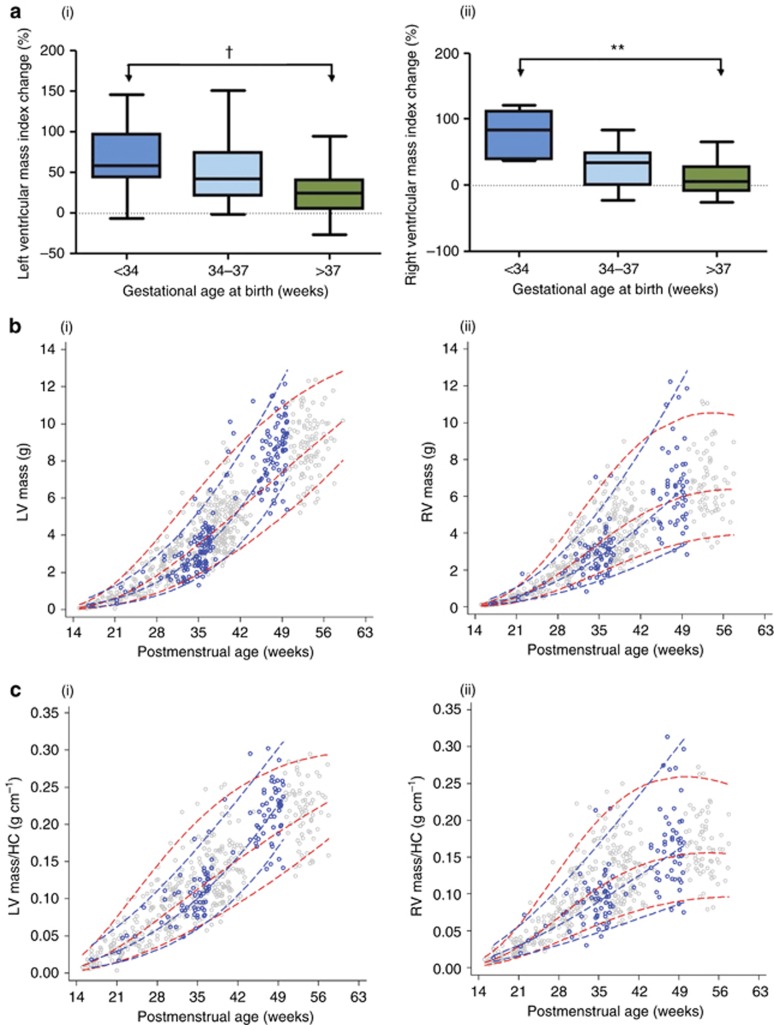
Ventricular mass in preterm versus term-born infants. (**a**) (i) Left and (ii) right ventricular mass index change between birth and 3 months increases with degree of prematurity (*P*-value relates to one-way ANOVA). (**b**) Trajectories of (i) left and (ii) right ventricular mass from 15 weeks of gestation through to 3 months of postnatal age for term (red with gray points) and preterm (blue with blue points) infants, and (**c**) trajectories indexed for head circumference demonstrate the preterm postnatal cardiac hypertrophy. Dashed lines indicate 3rd, 50^th^, and 97th centiles. LV indicates left ventricular; RV right ventricular; HC head circumference. **P<0.05; **P<0.01; ^†^*P*<0.001.

Infants born preterm had a smaller left ventricle (LV) mass and end-diastolic volume (EDV), indexed to body surface area, at birth compared with those born at term (Table 2). As the reduction was proportional, mass/EDV ratio was similar between groups, suggesting a smaller, but structurally similar, heart compared with those born at term. However, after 3 months, EDV index in those born preterm was similar to term-born infants, whereas left ventricular mass index, indexed to either body surface area or EDV, had become significantly greater (Table 2), with the percentage postnatal mass change in the preterm group being double that of the term group (change in LV mass index (LVMI) 57.8±41.9 vs. 27.3±29.4%, *P*<0.001). These volumetric mass changes were supported by the linear measures based on posterior wall thickness (PWd) and associated formulaic mass estimates (15) (Table 2), which also increased twofold compared with term counterparts (159.1±94.8 vs. 94.5±58.2%, *P*<0.001). Preterm neonates already had an increased right ventricle (RV) mass/EDV ratio at birth (Table 2) but also showed a twofold greater increase in RV mass index (39.3±38.1 vs. 16.6±40.8%, *P*=0.002). Changes in LVMI and RV mass index (RVMI) were proportional to gestational age at birth (for LVMI *r*=−0.49, *P*<0.001 and RVMI *r*=−0.37 *P*=0.001, Figure 2a), which was the main predictor of mass change in multivariate models that had included maternal pregnancy hypertension, birthweight *z*-score, 5 min Apgar score, and mode of delivery. In addition, although birthweight *z*-score was significantly positively correlated with gestational age, it was an independent predictor of LV mass change (Supplementary Tables S2 and S3). Similar patterns of mass change were seen in both genders. Interestingly, babies born by cesarean sections had lower Apgar scores across gestation (*P*<0.001) and for the preterm group alone (*P*=0.003). Both parameters were associated with mass change, although neither were significant in multivariable models (Supplementary Tables S2 and S3). Antenatal steroid exposure was excluded from multivariate regression models of LVMI and RVMI because of the significant co-linearity with prematurity. However, in a multivariate regression analysis with gestational age and antenatal steroids as the independent variables and change in LVMI as the dependent variable within the preterm group, gestational age remained highly significant (*P*=0.001), whereas exposure to antenatal steroids was not significant (*P*=0.67). Similarly, for RVMI, gestational age remained significant (*P*=0.03), whereas exposure to antenatal steroids was not significant (*P*=0.93). Furthermore, we have performed a subgroup analysis in our preterm group for LVMI and RVMI at birth and 3 months of postnatal age to compare those who were (*n*=93) and were not (*n*=28) exposed to antenatal steroids (Supplementary Table S4). These results indicate that there are no differences related to antenatal steroid exposure.

### Mass Change Relative to Normal Fetal to Neonatal Cardiac Growth Trajectories

We then compared cardiac mass in preterm infants with expected trajectories during the first 12 months of postmenstrual age. We overlaid the preterm trajectories onto one built from our fetal and neonatal echocardiographic measures from uncomplicated pregnancies (Figure 2b). All preterm measures at birth fell below the 95th centile for expected cardiac mass based on gestational age with mean *z*-scores within the group modeled from the expected cardiac fetal mass being −0.16±0.53 for the LV and −0.13±0.50 for the RV. By follow-up, however, several of the preterm infants were exceeding the 95% centile for cardiac mass with an absolute difference in the 50th centile at 49 weeks of postmenstrual age (3 months of postnatal age for a baby born at term) between preterm and term LV mass being 1.61 g. To allow for the change in body size during this period, we additionally created trajectories based on mass indexed to head circumference as a consistent measure between gestation and postnatal development (17) and demonstrated similar patterns (Figure 2c).

### Altered Left and Right Ventricular Systolic and Diastolic Function

**Figure 3:**
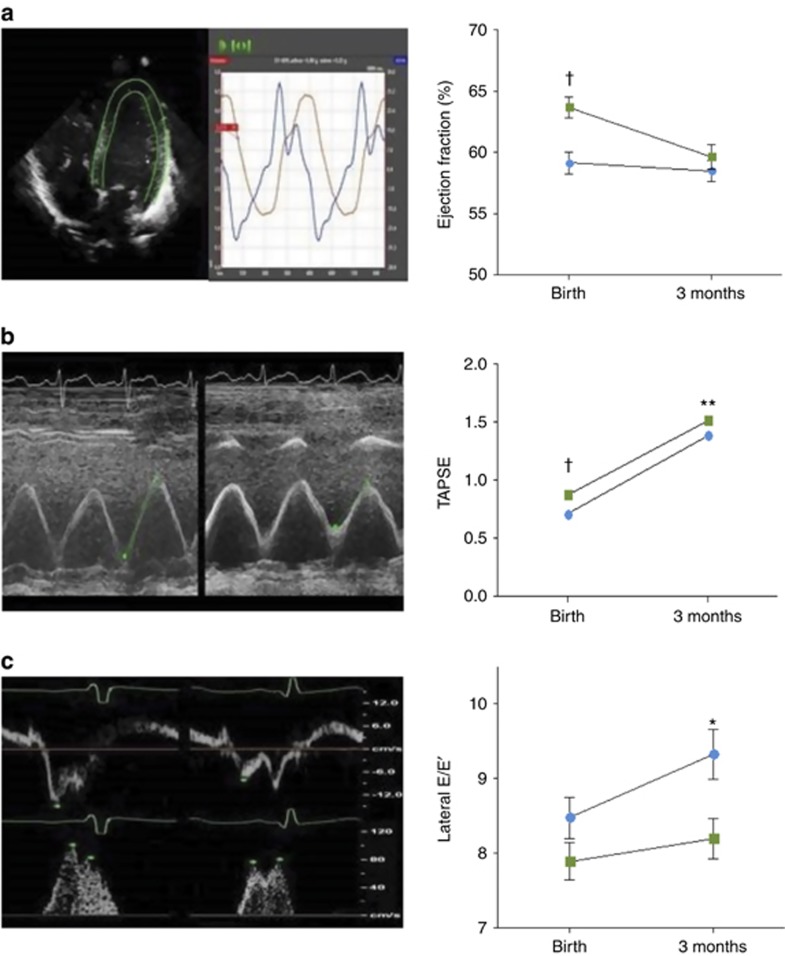
Ventricular function in preterm versus term-born infants. (**a**) Image demonstrates contoured four-chamber view on TomTec Image Arena 4.6 with figure that demonstrates a significantly higher ejection fraction in term (green) compared with preterm (blue) infants at birth that is no longer evident by 3 months of age. (**b**) Examples of M-mode measurements of tricuspid annular plane systolic excursion (TAPSE) that is significantly reduced in preterm (right) compared with term (left) infants at both birth and 3 months of age. (**c**) Examples of lateral mitral valve annular Tissue Doppler Imaging measures of early diastole velocities (*E*′) in term (left) and preterm (right) infants with corresponding pulsed-wave Doppler mitral valve inflow (*E*/*A* ratio). Lateral *E*′ is decreased and lateral *E*/*E*′ increased in preterm infants at 3 months of age. Error bars represent the standard error of the mean. *P<0.05; **P<0.01; ^†^*P*<0.001.

There was a reduced LV stroke volume and ejection fraction at birth in the preterm group related to both the reduction in EDV index and an increase in end-systolic volume (ESV) index. LV stroke volume, but not ejection fraction, remained reduced at 3 months of age (Table 2 and Figure 3a). In the right ventricle, RV systolic function, as measured by tricuspid annular plane systolic excursion (TAPSE), was reduced at both birth and 3 months in the preterm group (Table 2 and Figure 3b). At birth, there was a significant reduction in lateral *E*′ but not *E*/*E*′ in preterm infants, but by 3 months of age there was evidence of reduced relaxation with a significantly increased lateral *E*/*E*′ (Table 2 and Figure 3c). Interestingly, this increase was proportional to the increase in LVMI between birth and 3 months in this group (*r*=0.21 *P*=0.01).

### Shape Changes During Postnatal Development

**Figure 4:**
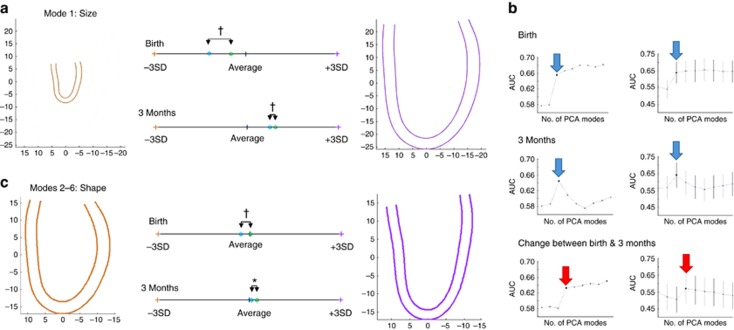
Ventricular shape in preterm versus term-born infants. (**a**) Significant shape differences between term (green) and preterm (blue) infants at birth are mainly accounted for by ventricular size (mode 1 in principal component analysis) with convergence by 3 months of age. (**b**) Linear discriminant analysis (LDA) identifies five further modes (modes 2–6), which account for the majority of shape variation independent of size between term and preterm infants. These describe variation between a “globular” and “conical”-shaped ventricle. Further modes did not significantly increase the area under the curve (AUC), confirmed by cross-validation (leave one out). Blue arrows indicate combination of modes 2–5 and red arrows 2–6. (**c**) Shape variations (mode 2–6) at birth between term and preterm infants persist after size adjustment and, again, are reduced by 3 months of age. Brown and purple contours demonstrate ±3 SDs from mean. Colored dots indicate relative placing of groups within shape range (term—green, preterm—blue). *P<0.05; **P<0.01; ^†^*P*<0.001.

Ventricular shape analysis suggested a unique preterm cardiac developmental pattern. The major mode that differentiated groups was, as expected, a general size mode (Figure 4a—mode 1). Therefore, to study specific shape changes, this mode was removed from analysis and linear discriminant analysis (LDA) was used based on the next five modes (modes 2–6). These accounted for the remainder of the variation between groups beyond which the addition of further modes did not increase the area under the curve for differentiation between groups. Modes 2–6 also all persisted in the cross-validation test (Figure 4b), and collectively they described variation between a ‘globular’ and a ‘conical’ heart (Figure 4c). At birth, the preterm heart tended to be more globular with a slightly narrower mitral annulus relative to mid-ventricular width (*P*<0.001), but this difference between groups had disappeared by 3 months of age (*P*=0.24).

## Discussion

This study shows for the first time that preterm infants have a greater increase in both LV and RV mass over the first three postnatal months that is disproportionate to increases in body and total cardiac size. Indeed, average cardiac mass changes are double that of their term-born counterparts during this period. Furthermore, there is a reduction in LV diastolic function with a persistent reduction in RV systolic function in the preterm infants. Finally, the hypertrophic patterns and global dysfunction are postnatal processes that are not evident during gestation or at birth, but rather emerges during the early postnatal period.

### Previous Studies

The increase in ventricular mass at 3 months of age is strikingly similar to our previous reports in adults born preterm (4, 5) and increased intraventricular septal thickness in children born preterm (22). Our longitudinal measures during postnatal development indicate that this pattern is not evident at birth but emerges during the early postnatal period. Some studies report ‘term-equivalent’ comparisons rather than post-delivery age, but we were able to model data from early gestation instead, which confirmed the postnatal pattern of increased mass. In fact, the 0.3 g difference in LV mass between groups at 3 months of age was substantially smaller than the 1.6 g difference between the two modeled 50th centiles at 49 postmenstrual weeks, suggesting that if we had used ‘term-equivalent’ ages, greater differences may have been seen between groups. However, longitudinal follow-up is required to confirm this.

Our observations are consistent with findings from a preterm-born sheep model, which had a five- to sevenfold increase in cardiomyocyte hypertrophy during postnatal development, as well as altered interstitial myocardial fibrosis and cardiomyocyte maturation (2). Similarly, a rat model of preterm birth conditions demonstrated increased left ventricular hypertrophy during the postnatal period and, furthermore, that these changes progressed to heart failure later in life when challenged with low-dose angiotensin II infusion and exposure to hypertension (3). Those born preterm display postnatal catch-up growth (23), but neither indexing for body size nor cardiac volume attenuated the large increase in cardiac mass in the preterm-born infants.

Our measures of mass at birth were similar to levels previously reported in babies of similar size, born at different gestations (24, 25, 26, 27), and in our preterm cohort were both proportional to ventricular size and within normal ranges for postmenstrual age on our fetal nomograms. Therefore, fetal influences on cardiac development in our preterm population are subtle. However, at birth, indexed LV mass tended to be slightly lower than that expected for postmenstrual age and compared with term-born infants, whereas indexed RV mass was slightly greater. This may relate to the use of indexing based on weight formulae, which avoid inaccuracies in length measurement, but tend to overcorrect in smaller infants (28). Alternatively, cardiac size differences may reflect the developmental stage of the preterm infant at birth. The right ventricle is dominant *in utero* and is thicker walled than the LV in fetuses up to 2.7 kg in weight (16, 29). Our preterm cohort had an average birthweight of 2.1 kg, and therefore may still have a more prominent RV. This could also explain the differences in cardiac shape at birth as, although globular-shaped LVs are associated with severe intrauterine growth restriction (30), this complication was not evident in our cohort. Shape changes had normalized by 3 months consistent with the known postnatal reduction in RV dominance. Our differences in TAPSE measures between preterm and term infants at birth and 3 months of postnatal age suggest reductions in RV longitudinal function (31). However, RV ejection fraction, which reflects global RV systolic function, did not differ between groups at either time point. It is possible that RV longitudinal functional changes emerge first, which may progress to global RV systolic reductions later in life (4).

### Influence of Other Perinatal Factors

Other perinatal factors linked with preterm birth might have influenced our findings. The use of antenatal steroids was not included in multivariable analyses because of the significant co-linearity with preterm birth. However, although we cannot be sure of an independent effect on mass, our results suggest that degree of prematurity, rather than antenatal steroids, has a more substantial contribution to the observed changes in cardiac mass. Multivariable analyses highlighted a small independent influence of birthweight *z*-score on the LV, consistent with a previous finding of mass increase proportional to birth size in very preterm infants (23). Maternal hypertension was associated with RV mass, which requires further investigation, but this effect was not significant in the multivariable model. The associations between mass, birth by cesarean section, and a low Apgar score are intriguing. Although, again, these variables were not significant in multivariable analyses, it might be hypothesized that these babies were exposed to *in utero* hypoxia, which has been shown to cause cardiac remodeling in animal models (32). Blood pressures in the cohort were appropriate for gestational age (33) and slightly lower at 3 months in the preterm group and therefore could not explain the hypertrophy. As previously observed (25, 34), we found that LV systolic function is reduced at birth in those born preterm but that LV ejection fraction had normalized by 3 months (35). The myocardium in preterm models has reduced myocardial contractile elements and inefficient myofibril shortening due to differences in calcium homeostasis (36). Therefore, it is possible that the increase in mass is partly a physiological compensation for reduced myocardial systolic performance (37).

Multiples were not excluded in this study. It is known that twins are at an increased risk of congenital heart disease, in particular in monozygotic pairs (38). Of the six preterm sets of twins, all were dizygotic except for one monozygotic pair. It is thus unlikely that these pairs would alter our findings. It is plausible that other known risk factors of cardiac hypertrophy that are more common in preterm infants born <32 weeks gestation, such as maternal diabetes, respiratory distress syndrome, and patent ductus arteriosus, would have led to even greater changes in cardiac mass. We were unable to assess the impact of these confounding factors given their low prevalence in our cohort of primarily moderate to late preterm infants. By studying primarily moderate and late preterm individuals, it allowed for a more direct assessment of preterm birth *per se* (31), and the findings are more widely applicable given that 80% of individuals are born between 32 and 36 weeks of gestation (39).

### Future Work

Longitudinal follow-up of this cohort will establish whether changes in mass and associations with function persist into later life. Furthermore, their relation to reduced exercise capacity (40) and higher blood pressures seen in those born preterm (41) will be established. If associations exist, then interventions including postnatal pharmacological approaches that improve myocardial development during this critical postnatal window, as demonstrated in animal models (42), may have long-term health benefits that could therefore be explored in future human studies. Postnatal nutrition, including exclusive human milk consumption and intravenous lipids, has already been shown to predict adult cardiac size and function (6, 7), although not cardiac hypertrophy, in those born preterm. It is possible that exclusive human milk diets may be a particularly beneficial area of research focus for improving cardiac development and reducing long-term risk in preterm offspring (6).

### Limitations

In the neonatal cohort, because of our stratified recruitment, the incidence of maternal hypertension was similar between groups (Table 1), which means that our term “control” group does not represent true normative data. However, it does limit the extent to which hypertension is acting as a confounding factor. In addition, because of the additional data sets from uncomplicated pregnancies in the fetal cohort, there was a greater proportion of hypertensive pregnancies in the preterm group when modeling cardiac growth trajectories, which needs to be taken into account during interpretation. Finally, only 55 infants had both antenatal and postnatal measurements performed when ideally each fetus should have been tracked across gestation and into postnatal development. However, data comparing the full and neonatal cohorts suggest minimal differences between them, and all mothers were selected from the same population setting.

Another potential limitation is our use of automated software to derive measures from single 4-chamber views. Neonatal and fetal echocardiography is technically challenging, although four-chamber views can be acquired in most fetuses by 13 weeks of gestation (43). Therefore, our use of a single plane increased data capture and allowed standardized, repeated measures from gestation through postnatal development. Assumptions about ventricular geometry means that absolute mass measures should be interpreted with caution. However, as similar assumptions were made across the cohort with good reproducibility, between-group differences are likely robust. Furthermore, our assumptions are supported by computational modeling of shape and findings replicated from linear wall thickness measures. The complex shape of the RV makes analysis difficult, but single plane measurements have been reported (16) and, as there are limited reports of RV changes, our findings add novel data to the literature.

### Conclusions

Preterm infants undergo a disproportionate increase in ventricular mass in the postnatal period, over and above what would be expected *in utero*, which is associated with cardiac dysfunction. These changes reflect those observed in experimental models of preterm delivery and in adults who were born preterm. The postnatal period appears to be a critical period of cardiac development and may offer a window for interventions to prevent long-term cardiovascular consequences of preterm birth.

## Figures and Tables

**Table 1:** Maternal, fetal, and postnatal characteristics of neonatal cohort

	Preterm (*n*=121)	Term (*n*=134)
*Maternal demographics and anthropometrics*		
Maternal age at delivery, mean (SD), years	33 (5.7)	32.3 (5.3)
Body mass index at booking, mean (SD), kg/m^2^	25.5 (5.1)	25.7 (6.8)
Smokers, *n* (%)	7 (5.8)	4 (3)
Maternal hypertension during pregnancy, *n* (%)	70 (58)	81 (60)
Maternal diabetes, *n* (%)	4 (3.3)	8 (6)

*Offspring birth characteristics*		
Gestational age at delivery, mean (SD), weeks	33.9 (2.2)	39.4 (1.3)
Male, *n* (%)	60 (50)	59 (44)
Birth order, median (IQ range)	1 (1)	1 (1)
Antenatal steroids, *n* (%)	93 (77)	1 (0.7)^†^
Cesarean section, *n* (%)	77 (64)	36 (27)
Apgar score at 5 min, median (IQ range)	10 (1)	10 (0)^†^
Birthweight, mean (SD), g	2053 (587)	3315 (563)^†^
Birthweight *z*-score, mean (SD)	−0.38 (1.1)	0.16 (1.1)^†^
Small for gestational age, *n* (%)	21 (17)	20 (15)
Days of ventilation, mean (SD)	0.63 (3.1)	0 (0)^†^
Ventilated/oxygen therapy for >28 days, *n* (%)	8 (6.6)	0 (0)^†^
Given surfactant, *n* (%)	7 (5.8)	0 (0)^†^
Treated patent ductus arteriosus (PDA), *n* (%)	3 (2.5)	0 (0)**
Sets of twins, *n* (%)	6 (9.2)	1 (1.5)**
		
*Offspring physiological measures at birth assessment*	(*n*=106)	(*n*=121)
Age at assessment, mean (SD), days	6.6 (5.4)	4 (5.5)^†^
Weight, mean (SD), g	2053 (587)	3315 (563)^†^
Body surface area, mean (SD), m^2^	0.16 (0.03)	0.23 (0.03)^†^
Head circumference, mean (SD), cm	30.7 (2.4)	34.5 (1.6) *
sBP, mean (SD), mm Hg	74.1 (14.7)	81.6 (13.4)^†^
dB, mean (SD), mm Hg	40.9 (9.9)	45.1 (9.4)^†^
		
*Offspring physiological measures at 3-month assessment*	(*n*=106)	(*n*=123)
Age at assessment, mean (SD), days	99.1 (15.1)	98 (13.8)
Weight, mean (SD), g	4960 (967)	6051 (894)^†^
Body surface area, mean (SD), m^2^	0.30 (0.04)	0.35 (0.04)^†^
Head circumference, mean (SD), cm	39.1 (2.1)	40.8 (1.7)^†^
sBP, mean (SD), mm Hg	93.7 (12.2)	96.8 (12.4)
dBP, mean (SD), mm Hg	50 (12.4)	54.4 (12.2)**

*P<0.05; **P<0.01; ^†^*P*<0.001 for comparison between term and preterm.

**Table 2:** Cardiac structure and function at birth and 3-month assessment

	Birth		Follow-up	
	Preterm	Term	Preterm	Term
*Left ventricle*				
Volumes	*n*=93	*n*=118	*n*=101	*n*=111
EDV, mean (SD), ml	2.7 (1)	4.2 (1.2)^†^	8.3 (2.1)	9.2 (2)**
EDV index, mean (SD), ml/m^2^	16.8 (5.5)	18.5 (4.3)**	27.6 (5.8)	26.8 (5)
ESV, mean (SD), ml	1.1 (0.4)	1.6 (0.7)^†^	3.5 (1)	3.7 (1)
ESV Index, mean (SD), ml/m^2^	7 (2.4)	6.8 (2.6)	11.7 (3.3)	10.9 (2.7)
				
Mass				
IVS diameter, mean (SD), cm	0.33 (0.08)	0.39 (0.08)^†^	0.44 (0.08)	0.43 (0.08)
PWd, mean (SD), cm	0.29 (0.06)	0.30 (0.07)	0.38 (0.07)	0.35 (0.06)**
Mass, mean (SD), g	3.1 (1)	4.7 (1.2)^†^	8.6 (1.7)	8.9 (1.8)
Mass Index, mean (SD), g/m^2^	18.8 (3.9)	20.7 (3.9)**	29.2 (6.5)	26 (4.8)^†^
Mass/EDV, mean (SD)	1.2 (0.3)	1.2 (0.2)	1.1 (0.3)	1 (0.2)**
Function				
Systolic function	*n*=72	*n*=95	*n*=62	*n*=66
Ejection fraction, mean (SD), %	59 (80)	64 (8)^†^	58 (7)	60 (8)
Stroke volume, mean (SD), ml	1.7 (0.7)	2.7 (0.7)^†^	4.8 (1.2)	5.7 (1.5)^†^
Diastolic function	*n*=95	*n*=122	*n*=90	*n*=109
EA, mean (SD)	1 (0.2)	1 (0.3)	1.1 (0.2)	1 (0.2)
Lateral *E*′, mean (SD)	6.1 (1.8)	6.6 (1.7)*	9.4 (2.3)	10.2 (2.2)*
Lateral *E*/*E*′ ratio, mean (SD)	8.5 (2.5)	7.9 (2.5)	10.1 (2.7)	9.4 (2.7)*
				
*Right ventricle*				
Volumes	*n*=51	*n*=76	*n*=67	*n*=75
EDV, mean (SD), ml	1.9 (0.9)	3.4 (1.5)^†^	4.7 (2)	5.3 (1.8)
EDV Index, mean (SD), ml/m^2^	11.1 (4.4)	14.7 (5.7)^†^	15.4 (5.7)	15.2 (4.4)
Mass				
Mass, mean (SD), g	2.8 (1.2)	4.1 (1.2)^†^	6.5 (2.2)	6.8 (1.8)
Mass Index, mean (SD), g/m^2^	16.3 (5.6)	17.9 (4.3)	21.5 (6.2)	19.3 (4.5)*
Mass/EDV, mean (SD)	1.6 (0.6)	1.4 (0.5)*	1.5 (0.5)	1.4 (0.6)*
Function	*n*=83	*n*=118	*n*=92	*n*=117
TAPSE, mean (SD)	0.7 (0.2)	0.9 (0.2)^†^	1.4 (0.3)	1.5 (0.3)**
Ejection fraction, mean (SD), %	49 (12)	51 (8)	56 (13)	56 (13)

*P<0.05; **P<0.01; ^†^*P*<0.001 for comparison between term and preterm at each time point.

## References

[bib1] Beck S, Wojdyla D, Say L et al, The worldwide incidence of preterm birth: a systematic review of maternal mortality and morbidity. Bull World Health Org 2010;88:31–8. 20428351 10.2471/BLT.08.062554 PMC2802437

[bib2] Bensley JG, Stacy VK, De Matteo R, Harding R, Black MJ. Cardiac remodelling as a result of pre-term birth: implications for future cardiovascular disease. Eur Heart J 2010;31:2058–66. 20453064 10.1093/eurheartj/ehq104

[bib3] Bertagnolli M, Huyard F, Cloutier A et al, Transient neonatal high oxygen exposure leads to early adult cardiac dysfunction, remodeling, and activation of the renin-angiotensin system. Hypertension 2014;63:143–150. 24166752 10.1161/HYPERTENSIONAHA.113.01760

[bib4] Lewandowski AJ, Bradlow WM, Augustine D et al, Right ventricular systolic dysfunction in young adults born preterm. Circulation 2013;128:713–20. 23940387 10.1161/CIRCULATIONAHA.113.002583

[bib5] Lewandowski AJ, Augustine D, Lamata P et al, Preterm heart in adult life: cardiovascular magnetic resonance reveals distinct differences in left ventricular mass, geometry, and function. Circulation 2013;127:197–206. 23224059 10.1161/CIRCULATIONAHA.112.126920

[bib6] Lewandowski AJ, Lamata P, Francis J et al, Breast milk consumption in preterm neonates and cardiac shape in adulthood. Pediatrics 2016;138:e20160050. 27302980 10.1542/peds.2016-0050 PMC6198929

[bib7] Lewandowski AJ, Lazdam M, Davis E et al, Short-term exposure to exogenous lipids in premature infants and long-term changes in aortic and cardiac function. Arterioscler Thromb Vasc Biol 2011;31:2125–35. 21817105 10.1161/ATVBAHA.111.227298

[bib8] Yu GZ, Aye CY, Lewandowski AJ et al, Association of maternal antiangiogenic profile at birth with early postnatal loss of microvascular density in offspring of hypertensive pregnancies. Hypertension 2016;68:749–59. 27456522 10.1161/HYPERTENSIONAHA.116.07586 PMC4978605

[bib9] Li F, Wang X, Capasso JM, Gerdes AM. Rapid transition of cardiac myocytes from hyperplasia to hypertrophy during postnatal development. J Mol Cell Cardiol 1996;28:1737–46. 8877783 10.1006/jmcc.1996.0163

[bib10] Campbell S, Thoms A. Ultrasound measurement of fetal head to abdomen circumference ratio in assessment of growth retardation. Brit J Obstet Gynaec 1977;84:165–74. 10.1111/j.1471-0528.1977.tb12550.x 843490

[bib11] Villar J, Ismail LC, Victora CG et al, International standards for newborn weight, length, and head circumference by gestational age and sex: the Newborn Cross-Sectional Study of the INTERGROWTH-21st Project. Lancet 2014;384:857–68. 25209487 10.1016/S0140-6736(14)60932-6

[bib12] Villar J, Giuliani F, Fenton TR et al, INTERGROWTH-21st very preterm size at birth reference charts. Lancet 2016;387:844–845. 26898853 10.1016/S0140-6736(16)00384-6

[bib13] Rychik J, Ayres N, Cuneo B et al, American Society of Echocardiography guidelines and standards for performance of the fetal echocardiogram. J Am Soc Echocardiogr 2004;17:803–10. 15220910 10.1016/j.echo.2004.04.011

[bib14] Leeson P, Augustine D, Mitchell ARJ, Becher H Echocardiography Second Edition. Oxford University Press: Oxford, 2012.

[bib15] Lang RM, Bierig M, Devereux RB et al, Recommendations for chamber quantification: a report from the American Society of Echocardiography's Guidelines and Standards Committee and the Chamber Quantification Writing Group, developed in conjunction with the European Association of Echocardiography, a branch of the European Society of Cardiology. J Am Soc Echocardiogr 2005;18:1440–63. 16376782 10.1016/j.echo.2005.10.005

[bib16] Ichihashi K, Ewert P, Welmitz G, Lange P. Changes in ventricular and muscle volumes of neonates. Pediatr Int 1999;41:8–12. 10200129 10.1046/j.1442-200x.1999.01008.x

[bib17] Hadlock FP, Deter RL, Harrist RB, Park SK. Fetal head circumference: relation to menstrual age. Am J Roentgenol 1982;138:649–53. 6978026 10.2214/ajr.138.4.649

[bib18] Boyd E. The Growth of the Surface Area of the Human Body. University of Minnesota Press: Minneapolis, 1935.

[bib19] Lamata P, Niederer S, Nordsletten D et al, An accurate, fast and robust method to generate patient-specific cubic Hermite meshes. Med Image Anal 2011;15:801–13. 21788150 10.1016/j.media.2011.06.010

[bib20] Lamata P, Sinclair M, Kerfoot E et al, An automatic service for the personalization of ventricular cardiac meshes. J R Soc Interface 2014;11:20131023. 24335562 10.1098/rsif.2013.1023 PMC3869175

[bib21] WHO Multicentre Growth Reference Study Group. Assessment of differences in linear growth among populations in the WHO Multicentre Growth Reference Study. Acta Paediatr Suppl 2006;450:56–65. 16817679 10.1111/j.1651-2227.2006.tb02376.x

[bib22] Mikkola K, Leipala J, Boldt T, Fellman V. Fetal growth restriction in preterm infants and cardiovascular function at five years of age. J Pediatr 2007;151:494–9. 17961692 10.1016/j.jpeds.2007.04.030

[bib23] Zecca E, Romagnoli C, Vento G, De Carolis MP, De Rosa G, Tortorolo G. Left ventricle dimensions in preterm infants during the first month of life. Eur J Pediatr 2001;160:227–30. 11317644 10.1007/s004310000702

[bib24] Walther FJ, Siassi B, King J, Wu PY. Echocardiographic measurements in normal preterm and term neonates. Acta Paediatr Scand 1986;75:563–8. 3751552 10.1111/j.1651-2227.1986.tb10251.x

[bib25] Ciccone MM, Scicchitano P, Zito A et al, Different functional cardiac characteristics observed in term/preterm neonates by echocardiography and tissue doppler imaging. Early Hum Dev 2011;87:555–8. 21576005 10.1016/j.earlhumdev.2011.04.012

[bib26] Guzeltas A, Eroglu AG. Reference values for echocardiographic measurements of healthy newborns. Cardiol Young 2012;22:152–7. 21933471 10.1017/S1047951111001259

[bib27] Kozak-Barany A, Jokinen E, Saraste M, Tuominen J, Valimaki I. Development of left ventricular systolic and diastolic function in preterm infants during the first month of life: a prospective follow-up study. J Pediatr 2001;139:539–45. 11598601 10.1067/mpd.2001.118199

[bib28] Ahn Y, Garruto RM. Estimations of body surface area in newborns. Acta Paediatr 2008;97:366–70. 18298786 10.1111/j.1651-2227.2008.00666.x

[bib29] Alvarez L, Aranega A, Saucedo R, Contreras JA. The quantitative anatomy of the normal human heart in fetal and perinatal life. Int J Cardiol 1987;17:57–72. 3666998 10.1016/0167-5273(87)90033-7

[bib30] Crispi F, Bijnens B, Figueras F et al, Fetal growth restriction results in remodeled and less efficient hearts in children. Circulation 2010;121:2427–36. 20497977 10.1161/CIRCULATIONAHA.110.937995

[bib31] Levy PT, Diodena B, Holland MR et al, Right ventricular function in preterm and term neonates: reference values for right ventricle areas and fractional area of change. J Am Soc Echocardiogr 2015;28:559–69. 25753503 10.1016/j.echo.2015.01.024 PMC4532398

[bib32] Tong W, Xue Q, Li Y, Zhang L. Maternal hypoxia alters matrix metalloproteinase expression patterns and causes cardiac remodeling in fetal and neonatal rats. Am J Physiol Heart Circ Physiol 2011;301:H2113–21. 21856922 10.1152/ajpheart.00356.2011 PMC3213965

[bib33] Zubrow AB, Hulman S, Kushner H, Falkner B. Determinants of blood pressure in infants admitted to neonatal intensive care units: a prospective multicenter study. Philadelphia Neonatal Blood Pressure Study Group. J Perinatol 1995;15:470–9. 8648456

[bib34] Koestenberger M, Nagel B, Ravekes W et al, Right ventricular performance in preterm and term neonates: reference values of the tricuspid annular peak systolic velocity measured by tissue Doppler imaging. Neonatology 2013;103:281–6. 23548493 10.1159/000348521

[bib35] Hirose A, Khoo NS, Aziz K et al, Evolution of left ventricular function in the preterm infant. J Am Soc Echocardiogr 2015;28:302–8. 25533193 10.1016/j.echo.2014.10.017

[bib36] Mahony L. Calcium homeostasis and control of contractility in the developing heart. Semin Perinatol 1996;20:510–9. 9090777 10.1016/s0146-0005(96)80065-6

[bib37] Anderson PA. The heart and development. Semin Perinatol 1996;20:482–509. 9090776 10.1016/s0146-0005(96)80064-4

[bib38] Best KE, Rankin J. Increased risk of congenital heart disease in twins in the North of England between 1998 and 2010. Heart 2015;101:1807. 26412859 10.1136/heartjnl-2015-307826 PMC4680160

[bib39] Ananth CV, Ananth CV, Vintzileos AM. Epidemiology of preterm birth and its clinical subtypes. J Matern Fetal Neonatal Med 2006;19:773–82. 17190687 10.1080/14767050600965882

[bib40] Svedenkrans J, Henckel E, Kowalski J, Norman M, Bohlin K. Long-term impact of preterm birth on exercise capacity in healthy young men: a National Population-Based Cohort Study. PLoS ONE 2013;8:e80869. 24324639 10.1371/journal.pone.0080869 PMC3855651

[bib41] Lewandowski AJ, Davis EF, Yu G et al, Elevated blood pressure in preterm-born offspring associates with a distinct antiangiogenic state and microvascular abnormalities in adult life. Hypertension 2015;65:607–14. 25534704 10.1161/HYPERTENSIONAHA.114.04662

[bib42] Bertagnolli M, Dios A, Beland-Bonenfant S et al, Activation of the cardiac renin–angiotensin system in high oxygen-exposed newborn rats: angiotensin receptor blockade prevents the developmental programming of cardiac dysfunction. Hypertension 2016;67:774–82. 26857347 10.1161/HYPERTENSIONAHA.115.06745

[bib43] Gembruch U, Shi C, Smrcek JM. Biometry of the fetal heart between 10 and 17 weeks of gestation. Fetal Diagn Ther 2000;15:20–31. 10705210 10.1159/000020970

