# Supplementary material for: Disproportionate cardiac hypertrophy during early postnatal development in infants born preterm
Source: Pediatr Res. 2017 May 24;82(1):36–46. doi: 10.1038/pr.2017.96 (PMC5511508; doi:10.1038/pr.2017.96)
Supplement: Supplementary Information [file pr201796x1.pdf]

Disproportionate Cardiac Hypertrophy During Early Postnatal Development in Infants Born Preterm

SUPPLEMENTAL MATERIAL

*Christina YL Aye [a,b], MA(Hons), DPhil, MRCOG; *Adam J Lewandowski[a], BSc(Hons), DPhil; Pablo Lamata[c], PhD; Ross Upton[a], MSc; Esther Davis[a], MBBS, DPhil; Eric O Ohuma[d,e], DPhil; Yvonne Kenworthy[a], BSc(Hons); Henry Boardman[a], MBBS, DPhil; Samuel Wopperer[a], BSc; Alice Packham[a], BA(Hons); Satish Adwani[f], FRCPCH; Kenny McCormick[f], FRCPCH; Aris T Papageorghiou[b], MD, FRCOG; Paul Leeson[a], PhD, FRCP

*Joint first authors. Both authors contributed equally to this work.

[a]Oxford Cardiovascular Clinical Research Facility, Division of Cardiovascular Medicine, University of Oxford, Oxfordshire, UK; [b]Nuffield Department of Obstetrics & Gynaecology, University of Oxford, Oxford, Oxfordshire, UK; [c]Department of Biomedical Engineering, King's College London, London, UK; [d]Nuffield Department of Medicine, Centre for Tropical Medicine and Global Health University of Oxford, Oxford, Oxfordshire, UK; [e]Centre for Statistics in Medicine, University of Oxford, Oxford, Oxfordshire,UK; and [f]Department of Paediatrics and Neonatology, John Radcliffe Hospital, Oxford, Oxfordshire, UK.

SUPPLEMENTAL METHODS

Clinical data collection

Characterisation of pregnancy complications and perinatal data related to the clinical care of the preterm infant were extracted from medical records retrospectively in a standardised way across studies. Preterm birth was defined as any birth before 37 weeks gestation. Hypertensive pregnancy diagnosis (pregnancy induced hypertension, preeclampsia) was defined according to ISSHP guidelines[1].

At birth and three months, three blood pressure measurements were recorded on the right calf, known to be comparable to arm measurements in neonates[2], with an automated digital monitor (Dinamap technology® V100) using appropriate sized cuffs and were averaged for analysis.

Image analysis

Quantification of cardiac mass and volume - Fetal loops were retrospectively gated offline using TomTec Image Arena 4.6 and end diastole was defined as the point of mitral valve closure. Neonatal loops were acquired with ECG tracing for cardiac gating. Mass and volume measures were obtained by manual contouring of the endo and epicardium using TomTec Image Arena 4.6 from the apical four chamber view for fetal, neonatal and infant echocardiography. The end diastolic frame was manually selected using the point of mitral valve closure as the marker and contours manually set at the inner endocardial edge and outer epicardial edge within the pericardium. To maximise reproducibility, the entirety of the septum was contoured for both left (LV) and right ventricular (RV) measurements. In addition, LV septal and posterior wall thicknesses in diastole were measured in the neonatal cohort from the parasternal long axis view in 2D using Philips Xcelera 3.3. The measurement was made at the base of the LV perpendicular to the mitral annulus through the leaflet tips.

To assess intra and inter-observer variability for measures, 10 datasets were selected at random (i.e. not selected for image quality) from fetal and neonatal datasets and reanalysed using the same set of selected cardiac cycles. Intraclass Correlation Coefficients (ICC) for single measures with 95% confidence intervals for intra and inter-observer variability yielded 1.00 (0.98-1.00) and 0.97 (0.83-0.99) for postnatal LV mass and 0.96 (0.84-0.99) and 0.81 (0.41-0.95) for postnatal RV mass respectively. ICCs for intra and inter-observer variability for fetal ventricular mass were 0.97 (0.87-0.99) and 0.81 (0.41-0.95) for the LV and 0.99 (0.95-1.00) and 0.78 (0.35-0.94) for the RV respectively.

Quantification of Ventricular Systolic and Diastolic Function – LV systolic parameters including ejection fraction, stroke volume and cardiac output were captured from automated tracking of the contours of the endocardium using TomTec Image Arena 4.6 as above. Manual adjustments of the contours were made, as required, throughout the cardiac cycle to ensure appropriate tracking of all segments and excluded if this was not possible due to image quality. RV systolic function was quantified by taking an M-Mode slice through the tricuspid annulus using the cursor in real time to measure the tricuspid annular plane systolic excursion (TAPSE), analysed offline using Philips Xcelera 3.3. Furthermore, RV ejection fraction was calculated from automated tracking of the contours of the endocardium using TomTec Image Arena 4.6. In the neonatal cohort at birth and three month follow-up, routine diastolic function parameters were assessed. Pulse Wave Doppler was measured from the mitral valve tips to assess early and late diastolic inflow and the ratio of these flows weas characterised as E/A ratio using Philips Xcelera 3.3. Further Doppler interrogation of the lateral mitral valve annulus using Tissue Doppler Imaging was measured in early diastole (E') and was utilised in the ratio of early diastolic flow to early diastolic tissue velocity (E/E') to assess myocardial relaxation in relation to the filling velocities. 10 datasets were again selected at random and the ICC for single measures with 95% confidence intervals for intra

and inter-observer variability were as follows: 0.99 (0.95-1.00) and 0.98 (0.94-1.00) for E/A ratio; 1.00 (1.00-1.00) and 0.93 (0.97-1.00) for lateral E'; 0.97 (0.87-0.99) and 0.90 (0.64-0.97) for TAPSE.

Morphology of the left ventricle – The endocardial and epicardial contours were exported from TomTec Image Arena 4.6 as text files that encode these contours and were fitted to a parametric description of a line. As a result, each contour was described with 52 coefficients, with each ventricle in the four chamber view therefore being reported using 104 coefficients (two contours, endocardium and epicardium). The left ventricles were aligned to their centre of mass and set in the vertical direction by the line that joins the centre of the base and the apex. The mean shape in the population was calculated and a Principal Component Analysis (PCA) of all cases was performed, finding the main modes of anatomical variation. The analysis was performed using a set of functions developed in Matlab for this purpose (Mathworks, Natick, Massachusetts, U.S.A.).

SUPPLEMENTAL RESULTS

Population characteristics

The mothers who delivered preterm had a similar body mass index (BMI) at pregnancy booking and prevalence of smoking. They were, however, on average a year older and were more likely to have had a hypertensive pregnancy disorder and a caesarean delivery ($p<0.001$). There was no bias by gender and birth order for term and preterm deliveries. Gestational age in the preterm group was six weeks younger than the term group (34.0±2.2 vs 39.7± 1.3 weeks) and they were significantly lighter at birth, with a lower birthweight z-score. Of the n=121 preterm individuals studied postnatally, 102/121 (84.3%) were born between 32-36 weeks gestation (moderate to late preterm); n=16 (13.2%) were born between

28-31 weeks gestation (very preterm); and n=3 (2.5%) were born less than 28 weeks gestation. Those with postnatal measures had similar characteristics to the full study group at birth [Supplementary Table 1] and the preterm group still had significantly lower weight and smaller head circumference at three months. Their systolic and diastolic blood pressures were also lower at birth, but by three months postnatal age, only diastolic blood pressure was lower. 337 fetal echocardiographic datasets were acquired, with 19 unanalysable for LV mass and 14 for RV mass. 229 echocardiography scans were performed at birth (18 unanalysable for LV and 100 for RV) and 227 echocardiography scans were performed at three months of age (14 unanalysable for LV and 100 for RV). 183 babies had LV mass measures and 81 had RV mass measures at both postnatal time points. For analysis of change in mass over the first three months of life, analysis was restricted to the group with measures at both time points. For trajectory changes, all analysable echocardiographic data were included and statistical approaches used as appropriate to allow for missing values in longitudinal datasets.

SUPPLEMENTARY TABLES

Supplementary Table 1: Comparison of Full and Neonatal Cohort Characteristics

	Full Cohort		Neonatal Cohort	
	**Preterm (n=128)**	**Term (n=264)**	**Preterm (n=121)**	**Term (n=134)**
**Maternal Demographics & Anthropometrics**				
Maternal age at delivery, mean (SD), years	32.7 (5.7)	31.7 (5.2)	33.0 (5.7)	32.3 (5.3)
BMI at booking, mean (SD), kg/m^2	25.4 (5.1)	24.9 (4.5)	25.5 (5.1)	25.7 (6.8)
Smokers, n (%)	11 (9)	22 (8)	7 (6)	4 (3)
Maternal hypertension during pregnancy, n (%)	70 (55)	99 (38)	70 (58)	81 (60)
**Offspring Birth Characteristics**				
Gestational age at delivery, mean (SD), weeks	34.0 (2.2)	39.7 (1.3)	33.9 (2.2)	39.4 (1.3)
Males, n (%)	65 (51)	124 (47)	60 (50)	59 (44)
Birth order, n (%)	1 (1)	1 (1)	1 (1)	1 (1)
Caesarean section, n (%)	78 (61)	59 (22)	77 (64)	36 (27)
Birthweight, mean (SD), grams	2074 (578)	3360 (507)	2053 (587)	3315 (563)
Birthweight z-score, mean (SD),	-0.38 (1.1)	0.20 (1.0)	-0.38 (1.1)	0.16 (1.1)

Supplementary Table 2: Bivariate Regression Coefficients for Maternal and Perinatal Characteristics and Change in Ventricular Mass

	**Change in Left Ventricular Mass Index (%)**		**Change in Right Ventricular Mass Index (%)**	
	**Unstandardised Coefficient (B)**	***P*-Value**	**Unstandardised Coefficient (B)**	***P*-Value**
**Maternal Factors**				
Age at delivery, years	0.70	0.20	-0.15	0.86
BMI at booking, k/m^2	0.03	0.95	0.57	0.50
Maternal smoking during pregnancy	16.87	0.34	-4.89	0.78
Maternal HTN during pregnancy	10.93	0.06	18.72	**0.04**
Maternal Diabetes	10.66	0.40	-24.58	**0.22**
**Perinatal Factors**				
Gestational age, weeks	-6.02	**<0.001**	-5.23	**0.001**
Caesarean section 4	19.56	**0.001**	25.67	**0.006**
Birthweight z-score	-9.53	**<0.001**	2.34	0.60
Sex	-3.11	0.59	8.01	0.38
Apgar score (5mins)	-7.49	**0.05**	-7.46	0.18
Antenatal steroid exposure	32.07	**<0.001**	33.45	**0.001**
Days of ventilation	12.24	0.19	*	*

*Not enough cases to compute coefficients
BMI indicates Body Mass Index; HTN hypertension

Supplementary Table 3: Multivariable Regression Coefficients for Maternal and Perinatal Characteristics and Change in Ventricular Mass

	**Unstandardised Coefficient (B)**	***P*-Value**
**Change in LVMI (%)**		
Gestational age, weeks	-5.08	**<0.001**
Caesarean section	-7.23	0.18
Birthweight z-score	-5.10	**0.03**
Apgar score (5mins)	-0.18	0.96
**Change in RVMI (%)**		
Gestational age, weeks	-4.41	**0.005**
Caesarean section	14.96	0.11
Maternal HTN	14.01	0.11

LVMI indicates left ventricular mass index; RVMI right ventricular mass index; HTN hypertension.

Supplementary Table 4: Left and Right Ventricular Mass Indexed to Body Surface Area at Birth and Three Months Postnatal Age in Preterm Individuals Exposed and Not Exposed to Antenatal Steroids

	**Steroids (n=93)**	**No Steroids (n=28)**	***P*-value**
LVMI at birth (SD), g/m^2	18.5 (3.6)	20.0 (4.7)	0.09
LVMI at follow up (SD), g/m^2	29.6 (6.9)	27.6 (4.0)	0.18
RVMI at birth (SD), g/m^2	16.0 (5.9)	17.3 (4.4)	0.48
RVMI at follow up (SD), g/m^2	21.6 (6.3)	21.2 (6.1)	0.80

LVMI indicates left ventricular mass index; RVMI, right ventricular mass index

REFERENCES

1. Brown MA, Lindheimer MD, de Swiet M, Van Assche A and Moutquin JM. The classification and diagnosis of the hypertensive disorders of pregnancy: statement from the International Society for the Study of Hypertension in Pregnancy (ISSHP). *Hypertension in pregnancy*. 2001;20:IX-XIV.
2. Park MK and Lee DH. Normative arm and calf blood pressure values in the newborn. *Pediatrics*. 1989;83:240-3.
